# Shaping the Genome with Non-Coding RNAs

**DOI:** 10.2174/138920211796429772

**Published:** 2011-08

**Authors:** Xue Q.D Wang, Jennifer L Crutchley, Josée Dostie

**Affiliations:** Department of Biochemistry, and Goodman Cancer Research Center, McGill University, 3655 Promenade Sir-William- Osler, Room 815A, Montréal, Québec, H3G1Y6, Canada

**Keywords:** Chromatin, epigenetics, gene regulation, non-coding RNA, polycomb repression complex, ribonucleoprotein complex, transcription.

## Abstract

The human genome must be tightly packaged in order to fit inside the nucleus of a cell. Genome organization is functional rather than random, which allows for the proper execution of gene expression programs and other biological processes. Recently, three-dimensional chromatin organization has emerged as an important transcriptional control mechanism. For example, enhancers were shown to regulate target genes by physically interacting with them regardless of their linear distance and even if located on different chromosomes. These chromatin contacts can be measured with the “chromosome conformation capture” (3C) technology and other 3C-related techniques. Given the recent innovation of 3C-derived approaches, it is not surprising that we still know very little about the structure of our genome at high-resolution. Even less well understood is whether there exist distinct types of chromatin contacts and importantly, what regulates them. A new form of regulation involving the expression of long non-coding RNAs (lncRNAs) was recently identified. lncRNAs are a very abundant class of non-coding RNAs that are often expressed in a tissue-specific manner. Although their different subcellular localizations point to their involvement in numerous cellular processes, it is clear that lncRNAs play an important role in regulating gene expression. How they control transcription however is mostly unknown. In this review, we provide an overview of known lncRNA transcription regulation activities. We also discuss potential mechanisms by which ncRNAs might exert three-dimensional transcriptional control and what recent studies have revealed about their role in shaping our genome.

## INTRODUCTION

Organisms with large genomes face the interesting problem of having to contain their genetic material within very small cellular volumes. For example, a human diploid genome measures approximately 2 meters in length and must fit within a micron-sized nucleus. Our genomic DNA must consequently be extensively packaged in a functional manner that allows activities like transcription, DNA replication and repair to occur properly. Although exactly how our genome is functionally organized *in vivo* is poorly understood, several key features appear common to most cell types (Fig. (**1**)). First, chromosomes are known to occupy distinct nuclear areas that are termed “chromosome territories” (CT). Gene-rich chromosomes tend to localize to the center of the nucleus and gene-poor near the periphery (reviewed in [1]). Genomic domains with similar activities and co-regulated genes often co-localize *in vivo* (reviewed in [2]). Moreover, genes located near anchor points where interphase chromosomes attach to the nuclear matrix tend to be poorly transcribed (reviewed in [3]). Together, these observations clearly reveal the existence of an intimate relationship between the organization of our genome in the nuclear space and gene activity. 

At the molecular level, spatial genome organization can critically affect gene expression. *In vivo*, genomic DNA exists in the form of chromatin where DNA is associated with histone and non-histone proteins. While it is well known that chromatin composition can directly shape gene activity [4, 5], three-dimensional chromatin organization is also emerging as an important gene regulation mechanism. For example, it was shown that distal control DNA elements could regulate target genes located on the same or on different chromosomes by physically interacting with them [6, 7]. This type of long-range regulation has been reported for genes located throughout the genome that are involved in various cellular pathways [8-12]. Consequently, the genome is now viewed as a functional network of physical contacts within (*cis*) and between (*trans*) chromosomes. These physical DNA contacts can be mapped at high-resolution with technologies such as the “chromosome conformation capture” (3C) and other 3C-related methods including 3C-Carbon Copy (5C) and Hi-C [13-17]. Given the recent innovation and sophistication of these approaches, it is not surprising that we still know very little about genome organization at the molecular level. In fact, most questions remain unanswered: How many types of contacts exist? What are their relative contributions to overall genome organization and to various genome functions? What are their tissue-specificities? What are their roles in the establishment and/or maintenance of cellular identity? What is their involvement in human disease? Answers to these questions will require extensive mapping and classification of chromatin interactions genome-wide under various cellular conditions. Only then will we be able to distinguish between regulated and structural contacts, and understand the outcome of altering either type on cellular function.

Even less well understood is what mediates chromatin interactions and how they are regulated. It was shown that chromatin contacts could be mediated by tissue-specific transcription factors. Such is the case for the beta-globin cluster where DNA looping between the locus control region (LCR) and activated beta-globin genes was shown to require the GATA-1 transcription factor and co-factor FOG-1 [18]. This type of long-range control mechanism where enhancers and distal promoters are brought in close physical proximity to each other by transcription factors has been found at other loci. These include many estrogen-regulated genes [19], the B lymphocyte-specific induction of *CIITA* by PU.1 [20], and *MYC* activation by TCF and the beta-catenin co-activator complex [21]. 

Other chromatin-binding proteins such as the CCCTC-binding factor (CTCF) and cohesin also appear to play critical roles in genome organization and gene expression [22, 23]. Mammalian CTCF is a DNA-binding protein associated with insulator sequences, boundary elements and imprinting control regions, all of which are thought to organize our genome into functional subdomains. CTCF is known to form networks of chromatin loops genome-wide and is viewed as a master regulator of spatial genome organization. It was also shown that CTCF co-localizes extensively with cohesin during interphase by directly binding to its STAG1/2 component [24]. Cohesin is a four protein subunit ring complex important in DNA repair, chromosome segregation and transcription regulation. Within some genomic regions, CTCF-co-localized cohesin rather than solely CTCF itself appears to be responsible for chromatin contacts. For example, cohesin knockdown was shown to abolish long-range *cis* chromatin contacts associated with *IFNG* locus expression in T helper 1 cells, and to reduce gene expression without affecting CTCF binding [25]. Similar cohesin-dependent loops and gene expression effects have also been observed at other loci. These include the beta-globin locus [26] and the apolipoprotein gene cluster [27]. Although they are often found together on the chromatin, CTCF and cohesin do not however exclusively co-localize with each other. Since cohesin does not bind DNA directly, this observation points to the existence of CTCF-independent cohesin recruitment and looping mechanisms. One such mechanism could involve tissue-specific transcription factors. Indeed, CTCF-independent cohesin binding sites frequently co-localize with tissue-specific transcription factors [28]. Alternatively, transcriptional coactivators such as mediator could recruit cohesin [29].

There are many ways by which chromatin interactions could be regulated. First, by modifying the DNA itself with cytosine methylation and consequently altering protein association. An example for this type of regulation is found at the imprinted *Igf2/H19* locus. On the maternal allele, expression of the *Igf2* gene is inhibited through the formation of a repressive loop encompassing the gene. This loop requires the recruitment of cohesin through CTCF bound at the DMR1 (DNA methylated region 1) and ICR (imprinted control region) sequences located upstream of *Igf2* and *H19* respectively [30]. Bound CTCF was also shown to recruit the polycomb repressive complex 2 (PRC2) at the *Igf2* promoter through its Suz12 subunit, thereby leading to allele-specific trimethylation of histone H3 lysine 27 (H3K27me3) and suppression of the maternal *Igf2* allele. In contrast, binding of CTCF to the ICR is negatively regulated by DNA methylation on the paternal allele. This lack of binding in turn prevents formation of the repressive loop, and enables access of the *Igf2* promoter to an enhancer located downstream of *H19* [31, 32]. 

Chromatin contacts could also be regulated by controlling access to DNA sequences with post-translational histone modifications (PTMs), the use of histone variants or by altering nucleosome positioning. Similarly, post-translational modification or changes in expression level of non-histone chromatin-binding proteins could represent important mechanisms to regulate chromatin contacts. Additionally, non-coding RNAs (ncRNAs) and their protein complexes could regulate the three-dimensional architecture of our genome. ncRNAs are a broad class of transcripts consisting of structural (rRNAs, tRNAs, snRNAs, snoRNAs, etc.), regulatory (miRNAs, piRNAs, etc.), and of sense/antisense transcripts, whose functions remain mostly uncharacterized. The latter RNA class includes transcriptional “features” (eRNAs, tiRNAs), and a very large number of long non-coding RNAs (lncRNAs) ranging in length from 200 nt to 100 kb. Although lncRNAs can be transcribed nearby known protein-coding genes or from their introns, they are often produced intergenically and hence, are sometimes referred to as “lincRNAs”. While a number of transcripts falling in that category have been studied for some time (e.g. Xist, see below), the realization that lncRNAs represent a very abundant RNA subclass is relatively recent and originates from genome-wide transcriptome studies [33-40]. lncRNAs are estimated to be at least as abundant as mRNAs, and like mRNAs, their expression levels and patterns are often tissue-specific. The heterogeneity in sequence, structure and size, along with the widely different subcellular localizations of lncRNAs points to their involvement in numerous cellular processes [41]. In the nucleus, a few lncRNAs were shown to inhibit or activate transcription, which is in contrast to other ncRNAs thought to represent basic features of transcription. 

Until recently, DNA sequence and associated proteins were thought to mainly dictate spatial genome organization. With the abundance and diversity of lncRNAs rivaling that of mRNAs, it seems likely that they also play an essential role in regulating chromatin organization. In fact, there is already evidence for the implication of ncRNA in general genome architecture [42], and in maintaining the integrity of nuclear bodies [43]. Conversely, chromatin conformation is thought to play a role in coordinating gene expression with lncRNAs [44]. Below, we report on the nuclear activity of a number of best-characterized lncRNAs, and discuss how lncRNAs might be instrumental in shaping the three-dimensional organization of our genome. 

## lncRNAs AS INHIBITORS OF TRANSCRIPTION

Although most lncRNAs have only recently been discovered, a few transcripts belonging to this category have already been scrutinized for some time. Amongst these, the X-inactive specific transcript (Xist) responsible for equalizing gene expression between mammalian males and females was first identified in the early 90’s [45, 46]. As described below, this lncRNA silences genes in *cis* by coating the chromosome. More recently, a lncRNA termed HOTAIR was shown to function in *trans.* Like the Xist transcript, HOTAIR associates with the polycomb repressive complex 2 (PRC2) and is thought to repress transcription by increasing H3K27me3 levels at specific genes. How these transcripts bind and help target polycomb complexes specifically to specific regions however is unknown. lncRNAs are also important for gene imprinting. For example, the Air lncRNA was shown to silence 3 genes in *cis* on the paternal allele at the *Kcnq-1* imprinted region. Like Xist, the Air transcript coats adjacent chromatin, but instead recruits the G9a histone 3 lysine 9 methyltransferase (H3K9me3) to repress transcription. Interestingly, other lncRNAs such as Kcnq1ot1 silence by binding both PRC2 and G9a to increase the levels of H3K9me3 and H3K27me3. Although repression by PRC2 binding and elevated H3K27me3 is thus far the most common mode of inhibition (see also ANRIL below), we can expect that many types of mechanisms will mediate lncRNA activity in *cis* and *trans* (Fig. (**2**)). In fact, alternative mechanisms have already been identified. For example, the p53-inducible lincp21 ncRNA was shown to bind hnRNP-K and inhibit gene expression genome-wide following p53 induction. We report below on the function and mechanism of some of these inhibitory lncRNAs. 

### Tsix Repression of Xist

In mammals, flies and worms, females differ from males by having two X chromosomes rather than one. The abundance of X-linked gene products must therefore be equalized between sexes in the somatic cells of these organisms. In mammals, gene dosage compensation is achieved by inactivating one X chromosome in females through a process called X chromosome inactivation (XCI). XCI requires the Xist lncRNA expressed in *cis*, which coats one female X chromosome and inactivates it by recruiting polycomb proteins (reviewed in [47]). Xist is expressed from a region on the X chromosome called X inactivation center (*Xic*) where its transcription is controlled by multiple lncRNAs. Indeed, the *Xic* encodes at least 6 other lncRNAs, 2 of which are known to antagonistically regulate Xist expression. On the one hand, the Tsix lncRNA transcribed antisense to *Xist* is known to repress Xist expression on the active X (Xa). Conversely, the Jpx lncRNA activates *Xist* on the inactive X (Xi). Tsix represses *Xist* transcription *via* multiple mechanisms that include recruiting DNA methyltransferase activity and the RNA interference machinery locally. Tsix is also thought to change the epigenetic chromatin state of *Xist*, thereby preventing Xist and RepA (a short transcript corresponding to the 5’end of Xist) from recruiting PRC2 (reviewed in [47]). Interestingly, the *Tsix* gene itself is positively regulated on Xa by another *Xic*-encoded lncRNAs called Xite. How Jpx regulates *Xist* will be described below in the next section. Together, XCI studies reveal that lncRNAs can regulate the transcription of other ncRNAs in addition to protein-coding genes. lncRNAs may therefore form very complex regulatory networks that control transcription genome-wide.

## HOTAIR

In 2007, Chang and colleagues reported the first comprehensive analysis of the transcriptional landscapes of the four human *HOX* clusters in 11 different fibroblast cell types [48]. In this study, 231 Hox ncRNAs were identified at 5-bp resolution on custom tilling arrays. Like *HOX* genes, these ncRNAs displayed differential expression along developmental axes and marked chromosomal domains with different histone methylation patterns and RNA pol II accessibility. The *HOX* clusters encode highly conserved eukaryotic transcription factors with pivotal roles in body patterning during development. *HOX* genes were originally identified in *Drosophila melanogaster* and their mammalian counterparts are known to have similar developmental functions. For example, studies in mouse models demonstrated that dysregulated *HOX* gene expression in early development could lead to severe limb and skeletal malformations [49-51]. *HOX* gene expression must also be controlled in adult tissues as overexpression of some *HOX* genes was found to be a hallmark of certain cancers such as leukemia [52, 53]. Although *HOX* regulation is clearly essential during development and for human health, how these genes are controlled remains poorly understood to this day. *HOX* clusters are regulated epigenetically and in pluripotent cells rest in so-called bi-valent domains where activating H3K4me3 and repressive H3K27me3 histone marks co-exist [54, 55]. The study led by Chang and colleagues uncovered not only a promising new level of *HOX* regulation, but also showed for the first time that lncRNAs can regulate genes in *trans*. Indeed, a lncRNA transcribed at a boundary between active and repressive chromatin states in the *HOXC* cluster was found to repress the expression of 5’ end *HOXD* genes located on another chromosome. This RNA is transcribed in an anti-sense manner to the *HOXC* genes and was named HOTAIR for “*HOX* Antisense Intergenic RNA”. HOTAIR is a 2,158 nt long spliced and polyadenylated RNA that binds PRC2 and silences genes by increasing H3K27me3 levels at specific sites [48, 56]. While the 5’ end of HOTAIR was shown to bind PRC2 directly through its Suz12 subunit, its 3’ end was also shown to bind the LSD1/CoREST/REST H3K4 demethylase complex. Thus, HOTAIR is thought to provide a scaffold for enzyme complexes that increase H3K27me3 (PRC2) and decrease H3K4me3 levels (LSD1/coREST/ REST) to silence gene expression [56]. 

In addition to the *HOX* genes themselves, which are involved in certain types of leukemia, Hox ncRNAs were also shown to be dysregulated in breast cancer [57]. Amongst these, high HOTAIR expression was found to correlate well with increased metastasis and lower patient life expectancy. Conversely, loss of HOTAIR expression reduced cancer invasiveness suggesting that lncRNAs, particularly HOTAIR, might significantly alter the epigenome of cancer cells. The association of lncRNAs with polycomb complexes sparks numerous questions: How can so many different lncRNA sequences bind directly to PRC2? How do these transcripts help target protein complexes to specific genes? Does the actual lncRNA sequence actively participate in targeting the silencing complexes to given genes? Might the local accumulation of lncRNAs around their transcription sites be sufficient to recruit histone-modifying enzymes and alter chromatin states? 

### Local INK4/ARF Locus Control by the ANRIL Non-Coding Transcript

The human *INK4/ARF* locus encodes three tumor suppressor genes named *p14^ARF^*, *p15^INK4B^,* and *p16^INK4A^* that are often altered in cancers [58]. In normal tissues, these genes are epigenetically repressed by PRC2-mediated deposition of H3K27me3, and are activated upon cellular stress to halt proliferation by inhibiting CDK4, CDK6 and MDM2. In 2007, a lncRNA transcribed anti-sense to the three tumor suppressor genes was identified along the *INK4/ARF* locus in human testes [59]. The mature 3,834-nt transcript was named ANRIL for “Antisense Non-coding RNA in the *INK4* Locus” and was derived from a 126 kb gene composed of 19 exons. In addition to full-length ANRIL, a shorter 2,659-nt form was also found in the same tissue, and 8 additional splice variants were later identified in several cell lines [60]. Although the roles of individual forms are unknown, full-length ANRIL was shown to repress *p15^INK4B^* expression [61]. Indeed, ANRIL knockdown in actively growing cells significantly increased expression of *p15^INK4B^*. Similarly, induction of *p15^INK4B^* and *p16^INK4A^* by oncogenic Ras was found to repress ANRIL expression. It was known that the three genes were silenced by PRC2 under normal cellular condition, and ANRIL knockdown significantly reduced PRC2 occupancy and H3K27me3 at the *INK4/ARF* locus. ANRIL was also found to co-immunoprecipitate with Suz12 and CBX7, which are components of PRC2 and the polycomb repressive complex 1 (PRC1), respectively [61, 62]. Like PRC2, PRC1 is a multisubunit protein complex and can bind H3K27me3 through its CBX components [63]. PRC1 is also responsible for the ubiquitination of H2AK119 known to inhibit RNA pol II transcription elongation [64-66]. Thus, the lncRNA ANRIL silences the *p15^INK4B^* gene by recruiting both polycomb repressive complexes to halt RNA pol II transcription elongation. 

Another anti-sense transcript named p15-antisense (p15AS) was identified at the *INK4/ARF* locus in 2008 [67]. Unlike ANRIL, this RNA measures 34.8 kb in length and overlaps only the *p15^INK4B^* gene. Consistent with the frequent epigenetic silencing of *p15^INK4B^* in leukemia patient samples, higher p15AS levels were often found in acute lymphoblastic (ALL) and myeloid leukemias (AML). Thus, it was suggested that p15AS could facilitate cancer progression by silencing the *p15^INK4B^* tumor suppressor gene. Surprisingly, p15AS overexpression increased the repressive H3K9me2 mark rather than H3K27me3, and decreased the levels of activating H3K4me2 near the TSS of the endogenous and exogenous *p15^INK4B^* gene. Similar histone modifications were also found at the *p15^INK4B^* promoter in cells where the gene is silent and no DNA methylation changes were observed. These results indicate that p15AS is capable of regulating its parental gene in* cis* and in* trans* through the formation of a type of heterochromatin. Although the silencing mechanism has not yet been identified, p15AS appears to trigger silencing but not maintain it since removal of the transcript did not reverse its effects.

### Gene Imprinting by Air and Kcnq1ot1

RNA transcripts important for gene imprinting were amongst the first lncRNAs identified in human and mouse. Gene imprinting is an epigenetic process that selectively represses one or more genes from a parental allele. This process was originally thought to result mainly from differential DNA methylation at CpG islands [68], but there is now overwhelming evidence that histone-modifying complexes and lncRNAs also play a significant role in this process. The Air and Kcnq1ot1 transcripts are two well-studied lncRNAs involved in gene imprinting [69, 70]. Interestingly, these transcripts differ from Xist, HOTAIR and ANRIL by their much larger sizes and absence of splicing [70, 71]. The Air transcript (antisense *Igf2r* RNA) is a 107,796-nt long antisense RNA transcribed from the second intron of the *Igf2r* gene to the 3’ end of the *Mas* gene [72]. Air is expressed only from the paternal allele where it silences the overlapping *Igf2r* gene and two other genes (*Slc22a2 and Slc22a3*) downstream of *Igf2r* [73]. The three genes are not consecutive and span approximately 300 kb indicating that Air acts specifically in *cis*. Although it is not spliced, Air is transcribed by RNA pol II and thus bears a 5' m(7)GpppN cap structure [74]. The full-length RNA transcript appears to be required for imprinting since deletion of its promoter or gene truncation prevented paternal silencing of the three genes [70]. Similarly to Xist, the Air lncRNA was found to coat its target genes but instead recruits the G9a histone methyltransferase rather than the subunits of PRC2 [75]. G9a is a SET domain-containing histone methyltransferase similar to polycomb Ezh2 but preferentially methylates H3 on lysine 9 (H3K9me3) [76-78]. Truncation substantially decreased Air lncRNA concentration around target genes, reduced G9a recruitment at the *Igf2r* locus, and failed to inhibit the *Igf2r*, *Slc22a2 and Slc22a3* genes [75].

The kcnq1ot1 transcript is another well-studied lncRNA involved in imprinting [79, 80]. Kcnq1ot1 (also called lit1 for “long QT intronic transcript 1”) is an antisense transcript that shares many common attributes with Air. First, it is a 91 kb RNA transcribed by RNA pol II, is capped, polyadenylated, but not spliced [71]. The transcript is expressed from an unmethylated promoter on the paternal allele of the *kcnq1* (potassium voltage-gated channel, KQT-like subfamily, member 1) gene and stays in the nucleus to silence protein coding genes in *cis* within the region [79, 81, 82]. In contrast to Air, which represses three genes at the *Igf2r* locus, Kcnq1ot1 can silence up to 10 genes in the *Kcnq1* cluster. Surprisingly, Kcnq1ot1 interacts with both G9a and the Ezh2 and Suz12 components of the PRC2 complex, and silences the surrounding genes by spreading H3K9me3 and H3K27me3 marks [71, 83]. A silencing domain was also identified at the 5’ end of the Kcnq1ot1 and further analysis revealed that Kcnq1ot1 silences gene expression not only by recruiting HMTs to remodel chromatin structure, but also by re-localizing its target genes near heterochromatin at the perinucleolar region [84].

### LincRNA-p21

The *p53* tumor suppressor gene encodes a key transcription factor, which is mutated in over 50% of human cancers. p53 is induced by many types of cellular stresses and exerts its tumor suppressor properties primarily through its ability to act as a sequence-specific transcription factor [85-87]. Many genes are upregulated or downregulated during the p53 transcriptional response. While p53-mediated gene activation is relatively well understood, how this transcription factor could lead to gene repression was unknown until recently. As lncRNAs had been shown to silence genes by recruiting chromatin-modifying complexes, it seemed possible that p53 might repress genes by activating lncRNAs. In 2010, Rinn and colleagues used high-resolution tiling arrays featuring predicted lncRNAs to probe the ncRNA transcriptome of p53-induced cells [88]. The custom tiling arrays included 400 genomic regions outside known protein-coding genes that were selected based on the presence of active H3K4me3 and H3K36me3 histone marks [89, 90]. Over 30 lncRNAs were induced by p53 in two different cellular systems indicating that like protein-coding genes, numerous lncRNAs are also temporally regulated during the p53 transcriptional response. Two lncRNAs containing canonical p53 binding sites in their promoters were shown to be *bona fide* p53 transcription targets and one was retained for further analysis. The lncRNA named lincRNA-p21 was interesting because of its curious genomic position approximately 15 kb upstream of the p21 cell cycle regulator, which is a well-known p53 target gene. lincRNA-p21 is a 3.1 kb spliced and polyadenylated RNA transcribed from the opposite strand to *p21*, and is conserved in human and mouse. Individual knockdown of p53 and lincRNA-p21 revealed a strong overlap in derepressed genes indicating that lincRNA-p21 participates in p53-mediated gene repression. Common derepressed genes were enriched in those involved in cell cycle arrest and apoptosis although further analysis indicated that lincRNA-p21 increases apoptosis without any significant effects on the cell cycle. Intriguingly, lincRNA-p21 was found to interact with hnRNP-K through a 780 nt region at its 5’end. Although deletion of this region abolished the ability of lincRNA-p21 to induce apoptosis, the region alone was not sufficient to trigger it. Furthermore hnRNP-K knockdown also showed a strong overlap in derepressed genes with lincRNA-p21 and p53, indicating that hnRNP-K is involved in the repression of many p53 downregulated genes likely through lincRNA-p21. Accordingly, the authors found that hnRNP-K is at the promoter of many p53-repressed genes by chromatin immunoprecipitation. How hnRNP-K can repress transcription however is unknown and it will be interesting to see how it is specifically recruited at gene promoters. 

## lncRNAs AS ACTIVATORS OF TRANSCRIPTION

Transcription activation by lncRNAs is a much more recent discovery than inhibition. Antisense ncRNAs transcribed intergenically from the *HOXA* cluster were amongst the first transcripts found to induce protein-coding genes. Like HOTAIR, *HOXA*-encoded lncRNAs are tissue-specific, transcribed by RNA pol II, spliced and polyadenylated. However, these transcripts appear to mainly induce the expression of neighboring genes in *cis*. In fact, no lncRNA has yet been shown to induce protein-coding genes in *trans*. Activating lncRNAs have since been reported genome-wide in a number of cell types. As for *HOXA* lncRNAs, they are often tissue-specific and transcribed by RNA pol II. However, some of these transcripts were found to induce distal genes within at least 300 kb away without necessarily affecting the ones adjacent to them. This type of specific long-range regulation suggests that three-dimensional chromatin architecture might be important for their activity. Activation by lncRNAs does not seem to be restricted to protein-coding genes since XCI was recently shown to require this type of mechanism. There will likely be many mechanisms by which lncRNAs can activate transcription (Fig. (**3**)) although most are currently unknown. Below are examples of lncRNAs thought to play a role in *cis* transcription activation. 

### Hox-Encoded ncRNAs, HOTAIRM1, and HOTTIP

An interesting aspect to *HOX* cluster regulation is reflected in the developmental expression pattern of their genes. During development, *HOX* genes are expressed in a spatio-temporal manner that is colinear with their positions along chromosomes [91, 92]. For example, genes located at the 3’ end of the clusters are usually expressed more anteriorly and earlier in the embryo than those at the 5’ end. Although genetic evidence suggests a role for three-dimensional chromatin organization in colinearity, how this type of *HOX* regulation occurs is unclear [93]. In early 2007, Orlando and colleagues used the well-described NT2/D1 cell differentiation system to investigate the role of intergenic *HOXA* ncRNAs in regulating *cis* gene expression [94]. NT2/D1 are human pluripotent embryonic carcinoma cells that can be terminally differentiated into neural-like cell lineages upon treatment with retinoic acid (RA). RA treatment also recapitulates the 3’-5’ induction pattern of the *HOXA1* to *A5* 3’ end genes in developing axial systems. The study showed that RA induces antisense *HOXA* ncRNAs in a manner that follows the collinear activation pattern of the protein-coding genes. Importantly, ncRNA induction was also accompanied by local changes in histone modifications and loss of repressive PcG complexes. It was proposed that antisense ncRNA transcription is fundamentally important to open and maintain the active state of *HOX* clusters during RA induction. In this model, the process of transcription itself rather than transcripts *per se* could represent an anti-silencing mechanism. The authors suggested that ncRNA genes might contain regulative epigenetic DNA elements and that transcription through them may counteract repression and maintain RA activation. Although we still do not know which elements could be at these sites, an attractive possibility is that transcription displaces chromatin-looping factors and disrupts repressive three-dimensional conformations. We previously reported the presence of long-range chromatin loops in the transcriptionally silent *HOXA* cluster in THP-1 and NT2/D1 cells [95, 96]. We found that RA treatment abolished these loops in NT2/D1 cells and suggested that the insulator-binding protein CTCF might be an important looping factor [96]. It will be interesting to determine the role of CTCF in regulating *HOXA* gene expression. 

*HOXA* ncRNA expression is not restricted to the induction of pluripotent cells with RA. *HOX* cluster transcriptome analysis in human fibroblasts actually identified hundreds of ncRNAs throughout the four clusters [48]. By using cells corresponding to various anatomical sites along developmental axes, this study revealed that ncRNA transcription is often tissue-specific and might demarcate silent and active chromatin domains. A tissue-specific antisense lncRNA transcribed between *HOXA1* and *A2* was more recently identified in the myeloid cell lineage [97]. Because of its myeloid-specific expression, the transcript was termed HOTAIRM1 for “*HOX* Antisense Intergenic RNA Myeloid 1”. HOTAIRM1 is 500 nt in length, transcribed by RNA pol II, spliced and polyadenylated. It is the most highly expressed *HOXA* lncRNA product following RA differentiation of NB4 cells into granulocytes and is highly expressed in leukocytes during normal human hematopoiesis. HOTAIRM1 knockdown in NB4 cells prevented RA induction of *HOXA1* and *A4*, and attenuated the induction of the *CD11b* and *CD18* granulocyte maturation genes. These results therefore suggest that transcription activation by HOTAIRM1 may not be restricted to its immediate *HOXA* neighboring genes, and it will be intriguing to determine its role in hematopoiesis and leukemogenesis. 

An article recently published by the Chang group also reports a lncRNA at the distal 5’ end of the *HOXA* cluster termed HOTTIP for “*HOXA* Transcript at the distal TIP” [44]. This 3,764-nt ncRNA is spliced, polyadenylated, and preferentially expressed at distal anatomical sites where 5’ end *HOX* genes are usually expressed. For example, HOTTIP was detected in human foreskin fibroblasts but not in lung. It was also expressed in mouse limbs at E13.5 and in chick limb buds. HOTTIP was shown to positively regulate the expression of 5’ *HOXA* genes in human distal fibroblasts and developing chick limbs. Indeed, knockdown of HOTTIP in foreskin fibroblasts resulted in lower 5’ *HOXA* gene expression and reduced H3K4me3 at promoters, but did not affect the levels of H3K27me3 along the cluster. Consistent with the role of 5’ end *HOXA* genes in limb development, HOTTIP shRNA injection in chicken embryos interfered with the formation of distal bony elements of the wing. These results show that HOTTIP might promote 5’ end *HOXA* transcription epigenetically by increasing the levels of activating histone marks. The mixed lineage leukemia proteins MLL1 and MLL2 are primarily responsible for trimethylation of H3K4 at the *HOX* clusters and HOTTIP was found to interact directly with the WDR5 component of the MLL modifying complex. HOTTIP knockdown reduced MLL-1 and WDR5 binding at the 5’ end *HOX* gene promoters suggesting that the lncRNA is required to recruit the methyltransferase complex to the chromatin. However, ectopic expression of HOTTIP in non-expressing cells did not activate the transcription of 5’ end *HOXA* genes. Similarly, HOTTIP overexpression in foreskin fibroblasts did not further enhance *HOXA* gene expression or rescue the effects of depleting endogenous nascent HOTTIP RNA. These results were explained with the suggestion that HOTTIP might only regulate genes when expressed immediately in *cis* of its targets and by hijacking pre-existing higher-order chromosomal structures that group regulated genes in close physical proximity to nascent HOTTIP RNA. Accordingly, 5C analysis of the *HOXA* cluster in fibroblasts indicated that HOTTIP expression correlates with enhanced chromatin interactions at the cluster 5’ end between *HOXA13* and *HOXA7*. Frequent chromatin contacts corresponded to higher H3K4me3 levels and low H3K27me3 marks, and HOTTIP knockdown had no effect on three-dimensional chromatin organization. Thus, HOTTIP might represent an example whereby spatial chromatin organization is required to regulate gene expression by lncRNAs in *cis*. We have previously shown that the transcriptionally silent *HOX* clusters are extensively folded in different cell lines [95, 96]. Our findings along with the *HOXA* structural data summarized above and the exquisite richness of *HOX* clusters in lncRNAs suggest that the clusters might be regulated in a cell type-specific manner by different mechanisms involving both chromatin architecture and lncRNAs. 

### Activating lncRNAs (ncRNA-a)

In 2010, Orom and colleagues used GENCODE annotation data to search for uncharacterized human lncRNAs [98, 99]. By focusing on intergenic transcripts classified to have no coding potential by the HAVANA group (Human and Vertebrate Analysis and Annotation; Sanger Institute), they identified 3019 putative long non-coding RNAs. These transcripts displayed chromatin signatures similar to protein-coding genes with high H3K4me3 at their 5’ ends and H3K36me3 along their bodies when transcriptionally active. In contrast to mRNAs however, the identified lncRNAs were shorter with an average transcript size of 800 nt. Their genes were also less complex than protein-coding genes with nearly 50% of them featuring a single intron. lncRNA expression analysis on custom arrays indicated that approximately 20% were ubiquitously expressed while over 33% displayed differential expression patterns across cell lines. Furthermore, it was found that the expression of many lncRNAs was regulated during cellular differentiation. Interestingly, functional knockdown analysis revealed an enhancer-like function for a set of lncRNAs. Indeed it was found that depletion of lncRNAs at multiple loci could result in a specific decrease in the expression of protein-coding genes in *cis*. Transcription activation was shown to be RNA-dependent, as evidenced by heterologous transcription assays. Importantly, some of these “ncRNA-a” were found to activate the transcription of critical regulators of development and cellular differentiation. For example, the Snai1 and Snai2 master regulators of hematopoiesis were downregulated following depletion of neighboring lncRNAs. Specifically, ncRNA-a7 RNAi knockdown was accompanied with less Snai1 expression and recapitulated the effects of Snai1 depletion itself. Interestingly, ncRNA-a7 depletion also led to a decrease in the expression of the *Aurora-kinase A* gene located 6 Mb away from the lncRNA gene. In fact, transcriptional effects were not restricted to immediate neighboring genes for many ncRNA-a, and could extend at least 300 kb away from the transcription units without necessarily affecting all genes in between. Thus, like *HOX*-encoded lncRNAs, these results suggest that spatial chromatin organization might play an important role in the specific activation of target genes. 

### Jpx Activation of Xist

In addition to activating the transcription of protein-coding genes, lncRNAs can also induce the expression of other lncRNAs. This type of regulation was found to be essential for XCI where Xist expression is repressed by Tsix on the Xa but induced by Jpx on Xi. Jpx is one of the 6 ncRNAs encoded at the Xic that was recently shown to be required for XCI [100]. Specifically, Jpx was found to derive mainly from the Xi and its expression to correlate with Xist transcription. Importantly, deleting Jpx in mouse blocked XCI and was female lethal. This phenotype was rescued by truncating Tsix suggesting that antagonistic lncRNA switches control XCI. Although it can work in *trans*, Jpx was found to have a mild preference for *cis* activation. Interestingly, previous 3C analysis of the Xic revealed higher-order chromatin contacts between the Jpx gene 5’ end and the Xist gene [101]. This result suggests that similarly to HOTTIP, three-dimensional chromatin organization might participate in preferentially targeting the Jpx lncRNA to its *cis*-located Xist allele. This study also reinforces an important principle in lncRNA function: that lncRNAs can regulate the expression of other lncRNAs. As such, it seems very likely that this type of regulatory network will play a role in the regulation of protein-coding genes.

## ncRNAs AS BASIC TRANSCRIPTIONAL FEATURES

Ascribing a function to long ncRNAs that are capped, spliced, polyadenylated and tissue-specific is intuitively easier to accept than to do the same for low abundance, short, and apparently unprocessed transcripts. In the past, short ncRNAs were often viewed as likely degradation products or as transcriptional “noise”, and were routinely discarded from datasets. Several groups have now taken a closer look at these RNAs and show that their non-random distribution is incompatible with aberrant transcription or with the truncated fragments of highly expressed genes. Short ncRNAs tend to be derived specifically from enhancer and promoter regions, and their expression levels, although very low, usually rises with increasing transcription (Fig. (**4**)). Regardless of whether these transcripts are simple manifestations of transcription activity or have actual regulatory functions, we can certainly learn much about transcription by taking a closer look at them. 

### Enhancer-Associated RNAs (eRNAs)

Enhancer sequences are scattered throughout mammalian genomes and are often activated in a tissue-specific manner. Enhancers can be identified by their association with the p300/CBP transcriptional co-activator, by the presence of histone 3 lysine 4 monomethylation marks (H3K4me1), and by their separate position to known transcription start sites (TSSs) [102]. Greenberg and colleagues recently used these enhancer traits to identify thousands of regulated enhancers during cortical neuron stimulation [103]. The study reports that most regulated p300/CBP binding sites are at least 1 kb away from known TSSs. Thus, transcription-dependent p300/CBP binding mainly occurs at enhancers rather than promoters in this system. Stimulation did not significantly change the histone modification landscape suggesting that chromatin is likely maintained permissive for induction in these cells. However, many transcription factors (TFs) were found to co-localize at enhancers, thereby raising the possibility that TFs might regulate enhancer function partly by recruiting p300/CBP. Strikingly, roughly 25% of enhancer-bound p300/CBP was found to bind RNA pol II in a transcription-dependent manner. Furthermore, short RNAs (< 2kb) transcribed bi-directionally from the center of enhancer domains could be detected from these sites. These transcripts were termed eRNAs for “enhancer RNAs” and appear to be non-polyadenylated. An interesting feature about eRNA transcription is that their levels correlate with the activity of nearby protein coding genes during neuronal stimulation. As such, enhancers may not strictly be recruitment sites of TFs, p300/CBP and RNA pol II but also sites of transcription. Changes in eRNA expression at actively transcribed enhancers were strongly correlated with regulated mRNA levels at nearby genes, and suggested that eRNA synthesis may only occur when enhancers interact with promoters. This hypothesis was confirmed by the absence of eRNA production from the active *Arc* gene enhancer in cells lacking the *Arc* promoter and most of the gene. Whether the transcriptional process itself or actual eRNA transcripts play a role in enhancer activation by modifying the chromatin state remains unknown. Nonetheless, this study not only clearly demonstrates that enhancers can be transcribed but also indicates that long-range looping rather than transcription-dependent RNA pol II tracking is a likely more widespread mechanism by which distal enhancers regulate target genes. 

### Transcription Initiation RNAs (tiRNAs)

Short ncRNAs mapping nearby gene promoters have been reported in several species over the past five years [104-106]. These studies together led to the hypothesis that genomes might encode multifunctional sequences that sometimes function as regulatory DNA elements and sometimes generate multiple independently regulated transcripts [107]. In 2009, the Mattick group examined small RNA deep-sequencing libraries from human, chicken and *Drosophila* to further characterize TSS-associated transcripts [108]. This study uncovered a new class of short ncRNAs mapping within 60 upstream to 120 bp downstream of annotated TSSs that were named tiRNAs for “transcription initiation RNAs”. The 5’ ends of tiRNAs peaked at 10 to 30 nts downstream from the TSS of their parent genes indicating that they are not simply RNA pol II runoff or truncated 5’ capped ends. They were found in all 3 species, and although slightly different in lengths, most were shorter than 22 nt with over a quarter measuring 18 nucleotides. tiRNAs were usually transcribed from the same strand as their corresponding TSS and were preferentially derived from GC-rich promoters. Consistently, promoters with tiRNAs were enriched in RNA pol II and binding of the Sp1 transcription factor. Curiously, tiRNAs were more frequently associated with highly expressed genes but their abundance did not directly correlate with gene expression levels. Furthermore, tiRNA synthesis was independent from the RNAi processing machinery indicating that they are likely derived directly from the RNA pol II activity at promoters. At least in *Drosophila*, tiRNA expression was only weakly associated with stalled transcripts produced at poised promoters. The authors therefore suggested that tiRNAs might represent products of RNA pol II backtracking, a process known to require the elongation and transcription cleavage factor TFIIS [109]. In a more recent paper, the same group reported a 40-fold nuclear enrichment of tiRNAs and demonstrated their association with transcription initiation marks [110]. Interestingly, tiRNAs were found to possess a 3’-guanine nucleotide bias in all 3 species, which further supports their non-random origin. tiRNA production is therefore a basic transcriptional feature in metazoans that may function in regulating chromatin modifications to fine-tune transcription. 

## lncRNAs AS REGULATORS OF SPATIAL GENOME ORGANIZATION

The histone-modifying activity of lncRNPs points to their pivotal role in regulating spatial genome organization. Although lncRNAs have thus far been predominantly associated with repressive complexes that methylate H3K27 or H3K9, it seems safe to predict that many more enzymatic activities will likely associate with these transcripts. Post-translational histone modifications may affect three-dimensional chromatin structure by altering the binding of nucleosomes to the DNA, the contacts between nucleosomes, or by changing the recruitment of non-histone proteins. For example, changes in histone charge introduced by acetylation or phosphorylation might significantly affect *in vivo* nucleosome interactions and chromatin compaction levels. Many protein complexes recognizing specific PTMs have now been identified and usually include enzymatic activities that further modify chromatin. Enzymatic activities include HDACs, HATs, HDMTs, HMTs, ubiquitin ligases, and chromatin remodelers. How histone PTMs can tether histone-modifying complexes has been extensively described elsewhere ([5] and references therein). 

At least in chicken and *Drosophila*, there is also evidence that ncRNAs themselves contribute to higher-order genome organization [42]. The Azorin group showed that polyA- RNA associates with chromatin where it represents approximately 2 to 5% of total nucleic acids. When they treated chromatin with RNase A, fragments sedimented less rapidly on sucrose gradients and were more sensitive to micrococcal nuclease. These structural changes were not correlated with any significant alteration in histone composition and were observed at euchromatin and heterochromatin. It was suggested that RNA might stabilize the binding of non-histone proteins to chromatin as was previously shown for HP1 at pericentric heterochromatin [111, 112]. A similar type of mechanism was also recently reported at repressed PcG target genes where short transcripts produced around TSSs can bind PRC2 complexes and might strengthen their association with the genes [113]. 

Specific ncRNAs may also nucleate the formation of functional nuclear subdomains or be required to maintain their integrity. The ncRNA NEAT1 (Nuclear Enriched Abundant Transcripts 1) is a very good example for this type of activity [114]. NEAT1 is a ubiquitously expressed nuclear lncRNA of 3.7 kb that is unspliced but polyadenylated [115]. It is highly expressed and localizes mainly to a type of membraneless nuclear body termed “paraspeckle”. The function of paraspeckles is not well defined but thought to represent nuclear retention sites of adenosine-to-inosine (A-I) edited transcripts [116]. NEAT1 knockdown was shown to disrupt paraspeckles and its overexpression to result in a larger number of them [117]. Another lncRNA termed NEAT2 for “Nuclear Enriched Abundant Transcript 2” was found to preferentially localize to splicing speckles, which is another type of nuclear subcompartment enriched in splicing factors [115]. NEAT2 is a 8.7 kb unspliced, polyadenylated transcript with a similar expression pattern to NEAT1 that represent the bulk of polyA+ RNA in speckles. Although NEAT2 does not appear to play a structural role in these bodies as detected by epifluorescence [117], there is evidence suggesting that it may regulate mRNA alternative splicing by altering the ratio of phosphorylated and unphosphorylated SR splicing factor inside the speckles [118]. 

In addition to their involvement in the function of nuclear subdomains, ncRNPs could also control higher-order chromatin architecture and transcription activity by mediating long-range contacts. There are many ways by which lncRNAs could affect gene expression through long-range interactions. For example, lncRNAs might cluster to form nuclear organization centers that coordinate gene expression. Good candidates for this type of activity are PcG-bound lncRNAs since polycomb complexes are known to form long-range *cis* and *trans* interactions genome-wide [119]. In this case, physical lncRNP networks would facilitate the coordinated down-regulation of multiple genes. Given the increasing complexity of RNA sequences found to interact with PRC2, it will be interesting to see which lncRNAs may be involved in this type of distal regulation. lncRNP-dependent physical networks could also be important for the formation of transcription factories (Fig. (**5A**)). Alternatively, lncRNA transcription might enhance or inhibit transcription of neighboring genes by displacing proteins from the chromatin and altering physical loops (Fig. (**5B**)). In this case, it is the transcription process itself rather than the transcripts that would mediate changes in gene expression. The lncRNAs genes themselves may also regulate chromatin architecture. For instance, lncRNA genes might cluster with other genes to promote the formation of transcription factories and gene expression. Additionally, lncRNA genes could compete with other genes (protein-coding or otherwise) for the same enhancers or other type of regulatory DNA element. 

## CONCLUSION

The discovery that lncRNAs represent a very large portion of our transcriptome has uncovered a new level of gene expression regulation. Although we know very little about them, their sequence diversity, potential splice variants, and cellular distribution suggests an important role in many aspects of RNA transcription, processing, and metabolism. The tissue-specificity and low conservation of lncRNAs amongst higher eukaryotes also points to their potential contribution in establishing or maintaining cell identity. Because of the overwhelming lncRNA complexity, recent studies have mainly focused on the function of individual transcripts in the nucleus. These studies clearly demonstrate that lncRNAs can regulate genes by various epigenetic mechanisms involving chromatin-modifying complexes. An interesting possibility is that lncRNAs also regulate transcription by altering chromatin architecture. Such spatial transcription regulation mechanisms could play an important role in human health and disease and it will be interesting to see how lncRNA function intersects with spatial chromatin architecture. 

## Figures and Tables

**Fig. (1).**
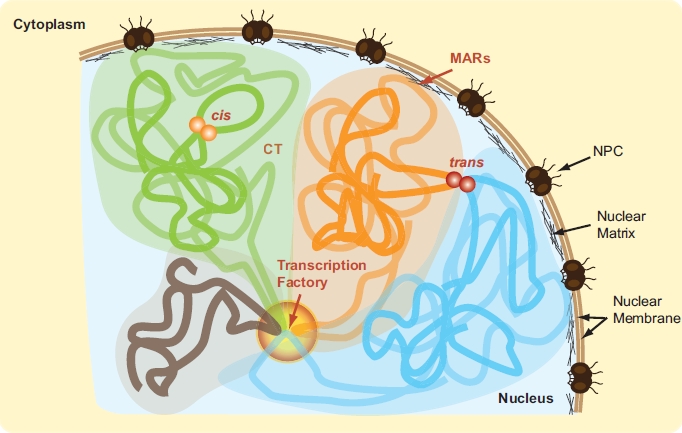
*In vivo genome organization.* Individual chromosomes are illustrated by thick green, orange, blue and grey strokes. Respective chromosome territories (CT) are highlighted in corresponding colors. Chromatin interactions within (*cis*) and between (*trans*) chromosomes are represented by orange and red spheres. Transcription factory is shown as a graded circle. MARs: matrix attachment regions. NPC: nuclear pore complex. (For interpretation of the references to color in this figure legend, the reader is referred to the web version of this paper).

**Fig. (2).**
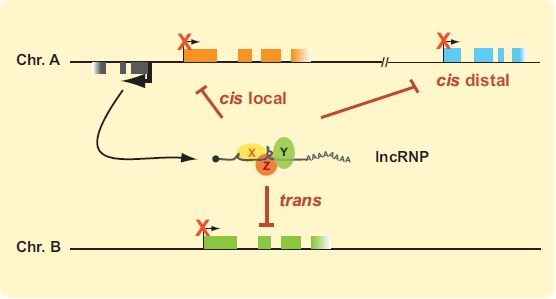
*lncRNAs as inhibitors of transcription.* lncRNAs expressed from anywhere in the genome can be packaged with transcript-specific proteins into functional lncRNPs (long non-coding ribonucleoproteins). The X, Y and Z ovals represent putative transcript-specific proteins associated with lncRNAs. lncRNPs could act locally or distally in *cis*, or in *trans*. Protein-coding genes are represented by orange, blue and green boxes. ncRNA gene is illustrated in grey. Transcription start sites (TSSs) are indicated by bent arrows, with thick arrows showing greater transcription activity. TSS of inhibited genes are indicated by thin bent arrows marked with an “X”. (For interpretation of the references to color in this figure legend, the reader is referred to the web version of this paper).

**Fig. (3).**
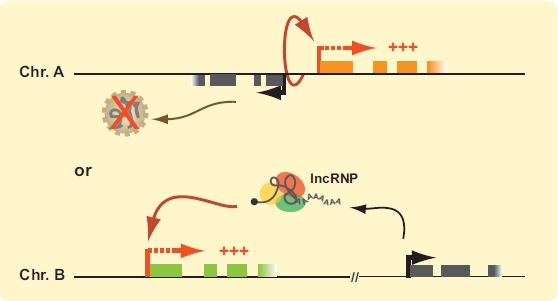
*lncRNAs as activators of transcription. (top)* The process of lncRNA transcription and not the transcript itself could be responsible for *cis* activation of neighboring genes by maintaining or initiating the chromatin structure in an active state. (*bottom*) lncRNAs can be packaged with transcript-specific proteins into lncRNPs to selectively activate gene expression in *cis*. Protein-coding genes are represented by orange and green boxes. lncRNA genes are illustrated in grey. Transcription start sites (TSSs) are indicated by bent arrows. Thick red bent arrows indicate active protein coding genes and thick black arrows active lncRNA genes. (For interpretation of the references to color in this figure legend, the reader is referred to the web version of this paper).

**Fig. (4).**
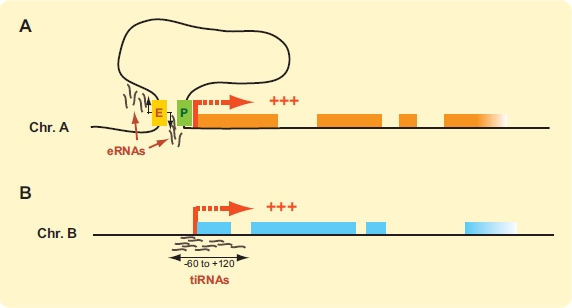
*ncRNAs as basic transcriptional features.* (**A**) Bi-directional short ncRNAs produced from the center of enhancer sequences can be detected in cortical neurons. These “enhancer RNAs” (eRNAs) are only found near active genes and require RNA pol II. eRNA production would presumably require contact between the enhancer and transcribed gene. (**B**) Short (18 nt) transcription initiation RNAs (tiRNAs) can be detected at active genes. tiRNAs preferentially map around TSSs of highly transcribed genes and sites of RNA pol II binding. Proteincoding genes are represented by orange or blue boxes. Active transcription start sites (TSSs) are indicated thick red bent arrows. Enhancer sequence (E) is shown as a yellow box and promoter (P) as a green rectangle. (For interpretation of the references to color in this figure legend, the reader is referred to the web version of this paper).

**Fig. (5).**
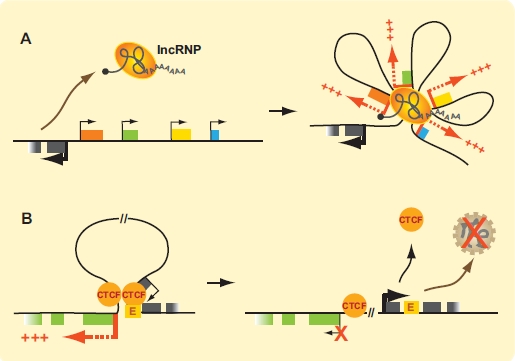
*lncRNAs as regulators of spatial genome organization.* (**A**) A lncRNP produced in *cis* or in *trans* could drive the formation of activating contacts that bridge enhancers to promoters, or induce the formation of a transcription factory. This example illustrates how a *cis* lncRNA could lead to the coordinated activation of gene clusters. (**B**) Activating long-range looping contacts between enhancers and promoters could be disrupted by transcription of a lncRNA. In this example, lncRNA transcription itself rather than the transcript could be responsible for transcription inhibition of neighboring genes. This example shows one CTCF protein displaced by transcription and disruption of an activating loop. Protein-coding genes are represented by colored boxes. Transcription start sites (TSSs) are indicated by bent arrows, with thick red bent arrows indicating active protein coding genes and thick black arrows active lncRNA genes. TSS of inhibited gene is indicated by a thin bent arrow marked with an “X”. (For interpretation of the references to color in this figure legend, the reader is referred to the web version of this paper).

## ABBREVIATIONS

3C: = Chromosome conformation capture
rRNAs: = Ribosomal RNAs
tRNAs: = Transfer RNAs
snRNAs: = Small nuclear RNAs
snoRNAs: = Small nucleolar RNAs
miRNAs: = MicroRNAs
piRNAs: = Piwi-interacting RNAs
eRNAs: = Enhancer RNAs
tiRNAs: = Transcription initiation RNAs
spliRNAs: = Splice-site RNAs
lincRNAs: = Long intergenic non-coding RNAs
lncRNPs: = Long non-coding ribonucleoprotein complexes
Igf2r: = Insulin-like growth factor II receptor
HMTs: = Histone methyl transferases
TSSs: = Transcriptional start sites
TFs: = Transcription factors
RNAi: = RNA interference
PTMs: = Post-translational modifications
